# Automated prostate tissue referencing for cancer detection and diagnosis

**DOI:** 10.1186/s12859-016-1086-6

**Published:** 2016-06-01

**Authors:** Jin Tae Kwak, Stephen M. Hewitt, André Alexander Kajdacsy-Balla, Saurabh Sinha, Rohit Bhargava

**Affiliations:** Department of Computer Science and Engineering, Sejong University, Seoul, 05006 Korea; Tissue Array Research Program, Laboratory of Pathology, Center for Cancer Research, National Cancer Institute, National Institutes of Health, Bethesda, MD 20850 USA; Department of Pathology, University of Illinois at Chicago, Chicago, IL 60612 USA; Department of Computer Science, University of Illinois at Urbana-Champaign, 2122 Siebel Center, 201 N. Goodwin Avenue, Urbana, IL 61801 USA; Beckman Institute for Advanced Science and Technology, Department of Bioengineering, Department of Mechanical Science and Engineering, Electrical and Computer Engineering, Chemical and Biomolecular Engineering and University of Illinois Cancer Center, University of Illinois at Urbana-Champaign, 4265 Beckman Institute 405 N. Mathews Avenue, Urbana, IL 61801 USA

**Keywords:** Prostate cancer, Database, Tissue morphology, Tissue retrieval, Infrared imaging, Decision support

## Abstract

**Background:**

The current practice of histopathology review is limited in speed and accuracy. The current diagnostic paradigm does not fully describe the complex and complicated patterns of cancer. To address these needs, we develop an automated and objective system that facilitates a comprehensive and easy information management and decision-making. We also develop a tissue similarity measure scheme to broaden our understanding of tissue characteristics.

**Results:**

The system includes a database of previously evaluated prostate tissue images, clinical information and a tissue retrieval process. In the system, a tissue is characterized by its morphology. The retrieval process seeks to find the closest matching cases with the tissue of interest. Moreover, we define 9 morphologic criteria by which a pathologist arrives at a histomorphologic diagnosis. Based on the 9 criteria, true tissue similarity is determined and serves as the gold standard of tissue retrieval. Here, we found a minimum of 4 and 3 matching cases, out of 5, for ~80 % and ~60 % of the queries when a match was defined as the tissue similarity score ≥5 and ≥6, respectively. We were also able to examine the relationship between tissues beyond the Gleason grading system due to the tissue similarity scoring system.

**Conclusions:**

Providing the closest matching cases and their clinical information with pathologists will help to conduct consistent and reliable diagnoses. Thus, we expect the system to facilitate quality maintenance and quality improvement of cancer pathology.

**Electronic supplementary material:**

The online version of this article (doi:10.1186/s12859-016-1086-6) contains supplementary material, which is available to authorized users.

## Background

Quality assurance in diagnostic histopathology plays a critical role in development of a treatment plan for patients with prostate cancer [1]. Methods to integrate quality development, maintenance, and improvement of diagnostic accuracy are, hence, critical to cancer management in any setting. In diagnostic prostate pathology, Gleason grading [2] is the most commonly used grading system that is based upon the structural patterns of the tumor. The Gleason grade is a primary determinant in treatment planning [3]. However, it is well known that the grading of prostate tissues suffers from intra- and inter-pathologist variability [4–6]; for example, the exact intra-pathologist agreement was achieved in 43–78 % of the instances, and 36–81 % of the exact inter-pathologist agreement was reported. It is also known that the variability of the grading can be reduced with focused retraining. There could be many ways to educate pathologists such as meetings, courses, online tutorials, and etc [7], but these are not time- and cost-effective and rarely implemented. Therefore, building an automated, fast, and objective method to aid pathologists in evaluating prostate can improve prostate cancer diagnosis.

When a pathologist evaluates a tissue sample, he/she looks at a stained tissue and mentally compares it against a fund of knowledge and experience and may consult publications when needed. In essence, the pathologist is matching structural patterns with samples they have seen earlier and mentally recalling the diagnosis made such that they can make the same diagnosis in the specific test case. Despite training, intra- and inter-observer variation and controversial areas still exist [8]. To aid and improve the diagnostic process, there have been several research efforts to develop automated systems for the detection and grading of prostate cancer. The majority of the previous methods have used morphological features [9–16] to characterize and classify tissue samples into correct classes, and others have also used Fourier Transform [17], Wavelet Transform [13, 18, 19], and Fractal Analysis [13, 20] to extract texture features. Though these methods claim to be accurate, the information that pathologists will obtain by using such methods may be limited since these only provide the predicted grade in general. The prediction also relies on the conditions of the training and testing datasets such as acquisition settings [15, 19] and staining [21].

Alternatively, content-based image retrieval (CBIR) systems [22–24] have been proposed to aid cancer pathology. The main objective is to effectively and efficiently manage an enormous amount of image data and to provide similar cases to a new test case that is examined. In addition to clinical usage, CBIR systems can help medical research, education, and training [22, 24]. The similar cases can be determined as owning the same grade [25–28] or sub-structures [29, 30]; for instance, single lumen glands, multi-lumen glands blood vessels, and lymphocytes in prostate [31]. The basic premise of such systems in diagnostic histopathology is that tissue samples that have the same grade or similar characteristics and patterns with the sample of interest will afford useful information to pathologists and improve the decision-making process. Similar to cancer detection and grading systems, tissue is represented as several quantitative features such as morphology [26, 32, 33], histogram [30], color [28, 34], and texture [27–29, 32–35]. The similar samples can be retrieved by computing distance metrics or similarity scores between a new case and the previously diagnosed or examined cases. In order to improve tissue representation and retrieval, features are often post-transformed and/or their weights are adjusted in an implicit or explicit manner; for example, kernel function [30], simplex method [32], manifold learning [26, 36], boosting [25, 27], and self-organizing map (SOM) [35].

Previous retrieval systems have been measured against a gold standard of diagnostic category and grade of tumor, defined by a pathologist. Prostate cancer is, in particular, a multifactorial disease and a mixture of heterogeneous growth patterns [37], and hence tissues belonging to the same Gleason grade may possess different cellular, nuclear, or glandular sub-patterns. A number of histological variants, in fact, exist in prostate carcinoma and some of the variants cannot be addressed by the Gleason grading system [38]. Moreover, the Gleason grading system results in a tumor grade that correlates with overall outcomes (survival), but fails to provide information on risk of metastasis, and correlates poorly with the clinical decision making process. Further, the Gleason grading system has gone through several refinements over time [8, 39–41] and may undergo further changes [42, 43]. These changes result variations among pathologists in practice [7] and disrupt developing robust automated grading and retrieval systems.

**Fig. 1:**
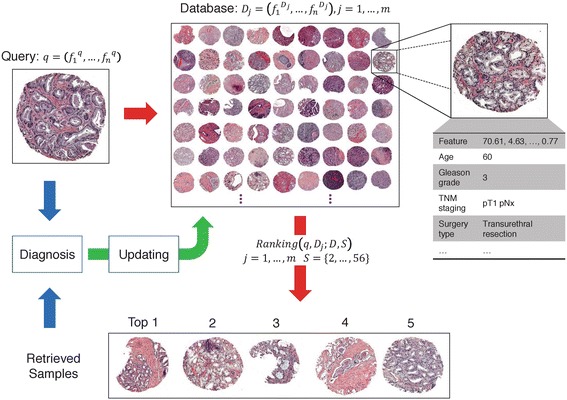
Overview of System. As a query is given, the closest matches and their clinical information are retrieved from the database (*red arrows*). Provided with the matching cases, pathologists make a diagnosis (*blue arrows*), and updating may or may not be conducted (*yellow arrow*). *Q*, *D*, *Ranking*, *f*, and *S* denote a query, database, retrieval process, single feature, and subset of features, respectively

Here, we report developing a new computer information and management and decision-support system that consists of a database of pre-defined prostate tissues and a retrieval process (Fig. 1). The database retains tissue images, clinical information, and one or more measurements of the structure of tissue. The retrieval process utilizes the structural/morphological features of the tissue sample image and provides tissue samples similar to the sample under consideration from the database. In assessing tissue morphology, we utilize infrared (IR) chemical imaging for providing cell type information in tissue [44]. We previously reported its utility in stabilizing and improving the automated cancer detection [45]. As a retrieval function, we adopt a machine learning ranking approach, called Ranking-support vector machine (Ranking-SVM) [46] in conjunction with a two-stage “feature selection” step [47]. Ranking-SVM learns a ranking function of high generalization due to maximum-margin property [48]. Feature selection step finds the most informative subsets while preserving the essential characteristics of the data. Moreover, we propose to determine the ground truth tissue similarity based on various structural properties of tissue, not solely on Gleason grade or a single structural component. Here, the structural properties are examined by pathologists. Combining different structural components of tissue ensures better characterization of tissue structure, and thus more accurate measurement of tissue similarity can be made. Thereby, the automated and computerized analysis and human experts’ assessment of tissue morphology are correlated through a machine learning approach.

The rest of the paper is organized as follows. In Methods section, we begin with a description of the dataset and data preparation process. In the following subsections, we describe the three key components of our new system – tissue similarity measure, tissue morphological feature extraction, and tissue retrieval function. Then, feature selection and balanced training are described. In Results section, the experimental results, including tissue similarity measure and tissue retrieval, via cross-validation are demonstrated. In Discussion section, the implications and limitations of our study are discussed. Finally, we conclude in Conclusions section.

## Methods

### Samples and data preparation

This study and protocols were approved by the University of Illinois Institutional Review Board (IRB) and was conducted as per the permission of the IRB in accordance with relevant guidelines and regulations. We have obtained 114 prostate cancer tissue samples (Tissue Array Research Program, National Cancer Institute and Clinomics Inc.), composed of 19 (Gleason 6), 26 (Gleason 7; 16 Gleason 3 + 4, 10 Gleason 4 + 3), 22 (Gleason 8), 10 (Gleason 9; 1 Gleason 4 + 5, 9 Gleason 5 + 4), and 37 (Gleason 10) samples. Both hematoxylin and eosin (H&E) stained and FT-IR images are available for the samples. Tissue samples were first sectioned to ~5um thick sections, with a section being placed on a standard glass slide and a serial section on IR transparent BaF_2_ slide. Stained with H&E, tissue images were acquired on a standard optical microscope at 40x magnification, and the size of a pixel is 0.963um × 0.963um. On IR transparent BaF_2_ slides, FT-IR images were acquired at a spatial pixel size of 6.25um × 6.25um and a spectral resolution of 4 cm^-1^ at an undersampling ratio of 2 using Perkin-Elmer Spotlight imaging system. The spectral profile of a pixel was truncated to a spectral range of 4000-720 cm^-1^. Detailed description of sample preparation and data acquisition for FT-IR imaging are available in Fernandez et al. [49]. Clinical information (Gleason grade, age, surgery type, etc.) of the samples were prepared by pathologic review, and 308 morphological features were also extracted. The database we build here, therefore, contains 114 tissue images (of two different modalities) and their clinical information and 308 morphological features.

### Morphologic criteria and tissue similarity measure

We define 9 criteria to describe the architectural properties of tissue: 1) Gland crowding, 2) Gland roundness, 3) Stromal reaction, 4) Nuclear grade, 5) Clefts 6) Lumen/gland ratio, 7) Gland continuity, 8) Cell separation, and 9) Gleason score. The details of the criteria are listed in Table 1. Some of the properties are the criteria used in the Gleason grading system, and others were adopted from the literature. Although some criteria are overlapped with the Gleason grading system, their importance and interpretation in our system may vary. The Gleason grading system may be also able to describe certain properties of tissues that cannot be characterized by the overlapping criteria. In the Gleason grading system, gland arrangement (Gland crowding), variations in size and shape of gland (Gland roundness), sheets of cells (Gland continuity), and single cells (Cell separation) are examined. Nuclear morphometry (Nuclear grade) [50–52], reactive stroma (Stromal reaction) [53–55], and retraction clefting (Clefts) [56] have been reported to be useful for prostate diagnosis and prognosis. In the digital and computerized tissue analyses, structural features describing gland arrangement [11, 36] and shape [11, 19, 36, 45] and the size of gland and lumen (Lumen/gland ratio) [12, 45, 57] have been adopted to characterize tissue. Individual cells also showed a moderate correlation with patient outcomes [58].

**Table 1:** Description of 9 Morphologic criteria

Criteria	Description	Score			
		0	1	2	3
Gland crowding	Gland tightness and cohesiveness	N/A	Sparse	Moderate	Very tight
Gland roundness	Roundness of external perimeter of gland	N/A	Very round	Moderate	Serrated contours or spindle shaped contours
Stromal reaction	Swollen, plump cells in stroma and splayed collagen fibers	N/A	No reaction	Little	-
Nuclear grade	Prominent nucleoli, variation in nuclear diameter and amount of chromatin	N/A	Normal	Some prominent nucleoli, moderate variation	Many prominent nucleoli, large variation
Clefts	Cleft formation or retraction artifact around cancer glands	N/A	<30 %	≥30 %	-
Lumen/gland ratio	Ratio between lumen area and total gland area	N/A	Wide lumen	Moderate lumen	Tiny lumen
Gland continuity	Continuous sheets of cells	N/A	<30 %	≥30 %	-
Cell separation	Individual cells separated by stroma	N/A	<10 %	≥10 %	-
Gleason Score	Predominant and secondary Gleason score	6 – 10^a^			

^a^Gleason score is the sum of predominant and secondary scores. In our set, it ranges from 6 to 10

For each of the criteria, a pathologist examines a tissue sample (H&E image) and assigns a score ranging from 0 to 2 (Stromal reaction, Clefts, Gland continuity, and Cell separation) or 0 to 3 (Gland crowding, Gland roundness, Nuclear grade, and Lumen/gland ratio) except Gleason score which in our set of tissues ranges from 6 to 10. The score range from 0 to 2 may be interpreted as none, low, and high, and the range from 0 to 3 may be considered as none, low, mid, and high. Due to its qualitative nature, it is difficult to highly stratify, and the impact and measurability of each criterion varies. Restricting the score range to none, low, mid, and high (or none, low, and high), in general, the scores are intended to be specific to differing morphologic patterns as well as to be reproducible by other pathologists. Using the scores of the 9 morphologic criteria, tissue morphologic similarity (*TMS*) between tissue samples is measured. Although well-defined and measured, the importance or relevance of each criterion differs. For example, the significance of Gland crowding score 1 may differ from that of Gland roundness score 1, and the difference between two samples having Gland crowding score 1 and 2 may not be identical to the difference between two samples owning Stromal reaction score 1 and 2. In these cases, the absolute values of the scores and the difference of the scores are identical. Recognizing the intrinsic relationship between scores and criteria, we utilize the distribution of each criterion score over the samples in the database. Regardless of the absolute value of a score, two samples away from each other in the distribution of the scores of a criterion are likely dissimilar in terms of the criterion, and vice versa. In other words, tissue similarity between two samples with respect to a morphologic criterion is related to the number of samples between them as ordered by the score for the criterion. Accordingly, let *TMS*(*d*_1_, *d*_2_) be the tissue morphologic similarity between two tissue samples *d*_1_ and *d*_2_ and computed as follows:

$$ TMS\left({d}_1,{d}_2\right)={\displaystyle {\sum}_{i=1}^9TM{S}^i\left({d}_1,{d}_2\right)} $$

where *TMS*^*i*^(*d*_1_, *d*_2_) is the morphologic tissue similarity for *i*th criteria. *TMS*^*i*^(*d*_1_, *d*_2_) is calculated as follows:

$$ TM{S}^i\left({d}_1,{d}_2\right)=1-\frac{{\displaystyle {\sum}_{s={s}_{d_1}^i+1}^{s_{d_2}^i-1}{h}^i(s)}+\frac{1}{2}\left({h}^i\left({s}_{d_1}^i\right)+{h}^i\left({s}_{d_2}^i\right)\right)}{Z} $$

where *s*_*d*_^*i*^ is the *i*th morphologic criterion score of a tissue sample *d*, *h*^*i*^(*s*) is the number of samples having *i*th morphologic criterion score *s*, and *Z* is a normalization factor. Due to normalization, *TMS*^*i*^(*d*_1_, *d*_2_) ranges from 0 to 1, 1 ≤ *i* ≤ 9, thereby *TMS*(*d*_1_, *d*_2_) ranges from 0 to 9. In this study, *TMS* scores represent the true similarity between tissue samples and serve as the gold standard of tissue retrieval.

### Morphological feature extraction

**Fig. 2:**
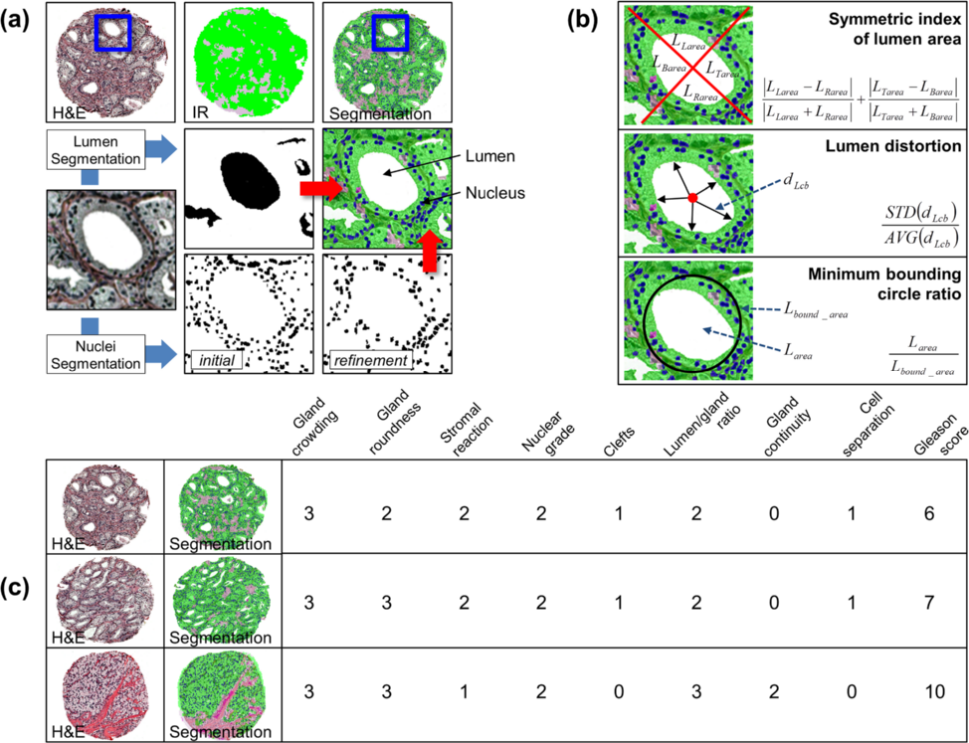
Morphologic Feature Extraction and Morphologic Criteria. **a** Cell type segmentation from FT-IR imaging is overlaid with a tissue image (H&E). Lumen (*white*) and nuclei (*blue*) are segmented using tresholding and applying shape, size, and intensity constraints. **b** Using the segmentation results, a number of morphological features are computed. **c** A pathologist examines and scores tissue images (H&E) for the 9 morphologic criteria. The segmented tissue images are also shown for comparison

In prostate cancer, epithelial cells [59], which line ducts and acini in intact tissue in three-dimensional structures, are of great interest. As cancer grows, epithelial cells grow (or invade) in and out of the glands in an uncontrolled way, and thus the structure of tissue, especially the local glandular structure, is distorted. We also note that the role of stroma cells, connective cells supporting epithelial cells, in cancer tissue has been recently recognized [53, 54]. To quantify the alterations in tissue morphology, we focus here on the nuclear and cellular morphology of epithelial and stromal cells and lumens (empty space inside a gland). In order to quantify the nuclear and cellular morphology of epithelial and stromal cells and lumens (Fig. 2a), we first segment epithelium and stroma in tissue by adopting Fourier transform infrared (FT-IR) spectroscopy imaging due to its accuracy and robustness [44]. FT-IR has been extensively validated in classifying histologic cell types in tissue [49, 60, 61] and provides a color coded cell type image of tissue. Cell type segmentation in H&E images is challenging due to limited information, color variations, etc. Rigid-body image registration overlays the epithelium and stroma segmentation from FT-IR imaging with the corresponding H&E image by using outer shape and empty space (lumens) in tissues [45]. Second, lumens and nuclei are identified from H&E images by considering their color intensities and geometric properties [45]. Using the segmented nuclei and lumens, we finally define a number of quantities measuring the morphologic changes in tissue, and the quantities include the size, number, distance, spatial distribution, and shape of epithelial nuclei and lumens (Fig. 2b). Detailed description of the quantities is available in Supplementary Information. In total, we defined 26 quantities, of which 17 quantities were previously shown to be effective in detecting prostate cancer tissue with high accuracy [45]. Computing average, standard deviation, sum total, minimum, and maximum of all or some of these quantities, 308 morphological features are extracted from a tissue sample. The details of tissue segmentation and feature extraction process are described elsewhere in Kwak et al. [45].

### Tissue retrieval

Given a query (unknown tissue sample image), its morphological features are extracted and used to search for similar pre-examined samples from the database. To retrieve the most similar samples, we adopt Ranking-SVM [46], which learns a function mapping onto a ranking in a pair-wise fashion (see Supplementary information for details). That is, Ranking-SVM provides a complete ranking of the entire samples in the database for the query. Since *TMS* score serves as the gold standard of the tissue similarity (or ranking), Ranking-SVM attempts to learn and reproduce the human experts’ interpretation of the tissue samples. The feature vector difference between a query and the samples in the database is used for retrieval. We note that if a sample in the database is highly ranked to the query, then the query should be highly ranked for the sample (if we switch the highly ranked sample with the query). Ranking-SVM is an asymmetric measure, i.e., the ranking of a sample to the query would not be equal to the ranking of the query to the sample. Combining the two rankings, we seek to attain the more symmetric rankings between the query and the samples and to achieve the more accurate and specific retrieval (the samples that are similar to both the query and other samples in the database will be penalized, and the samples that are similar to the query and dissimilar to others will be boosted). We define the ranking of a sample to the query as

$$ Ranking\left(q,{d}_i;D\right)= Ranking-SVM\left(q,{d}_i;D\right)+ Ranking-SVM\left({d}_i,q;D\backslash {d}_i\cup q\right),\ i=1,\dots, m $$

where *Ranking* ‐ *SVM*(*q*, *d*_*i*_; *D*) denotes the ranking of the sample *d*_*i*_ in the database *D* to the query *q* and *Ranking* − *SVM*(*d*_*i*_, *q*; *D*\*d*_*i*_ ∪ *q*) is the ranking of the query *q* to the sample *d*_*i*_ in the database D when the query *q* is switched with the sample *d*_*i*_. Based on the ranking, Top-*T* samples are retrieved. Since it is the sum of two rankings, it is likely that several rankings are tied. In such cases, the final ranking is determined by the ranking of the sample to the query, i.e., *Ranking* − *SVM*(*q*, *d*_*i*_; *D*), which is intuitive because the retrieval is done for the query.

### Feature selection

Feature selection is the step where the retrieval algorithm examines all available features (308 in our case) with respect to the training samples, and selects a subset to use on test data. This selection is generally based on the criterion of high accuracy on training data, but also strives to ensure generalizability beyond the training data. We adopt a two-stage feature selection approach here. In the first stage, we order the features by their individual retrieval performance and sequentially measure the retrieval performance of a feature set by adding a new feature one at a time according to the order. In the second stage, feature selection continues with the feature set resulting the best retrieval performance in the first stage as the starting point, following the sequential floating forward selection (SFFS) method [62]. This method sequentially adds new features followed by conditional deletion(s) of already selected features.

Throughout the feature selection procedure, the retrieval capability of a feature set is measured by normalized discounted cumulative gain (NDCG) [63, 64], which is a popular measure to evaluate ranking algorithms with multiple levels of relevance. NDCG utilizes the relevance (*TMS* score in our study) and ranking of the retrieved samples and is based on two assumptions: 1) highly relevant samples are more valuable when they are retrieved earlier 2) highly relevant samples are more valuable than marginally relevant samples to the query. Given a database *D* and *TMS* scores, the performance of the retrieval function *f* for a query *q* at rank position *T* is computed as follows:

$$ NDCG\left(q,f;D,TMS\right)=\frac{DCG}{IDCG} $$

$$ DCG\left(q,f;D,TMS\right)={\displaystyle {\sum}_{t=1}^T\frac{2^{TMS\left(q,{r}_t\right)}-1}{{ \log}_2\left(1+t\right)}} $$

where *r*_*t*_ indicates the *t*th closest sample to the query *q*, retrieved by the retrieval function *f*, from the database *D*, and *IDCG* denotes a normalization factor that is computed with the ideal (or optimal) rank of the retrieved samples, scaling the optimal retrieval to 1.

### Balanced training

Ranking-SVM tries to learn an overall ranking of the training dataset. When trained on biased or unbalanced training dataset, Ranking-SVM may be biased towards dominant dataset, and thus its retrieval capability may be limited. To prevent this, we sought to take roughly balanced sub-samples of the training dataset and trained Ranking-SVM on the sub-samples. To obtain the roughly balanced training dataset, we first divide the total *TMS* score range into *P* equal-width partitions. Then, *N*_*P*_ number of pairs of samples from each partition was randomly selected. We set *N*_*P*_ to the smallest number of pairs of samples in a partition.

## Results

### Tissue morphologic similarity measure

**Fig. 3:**
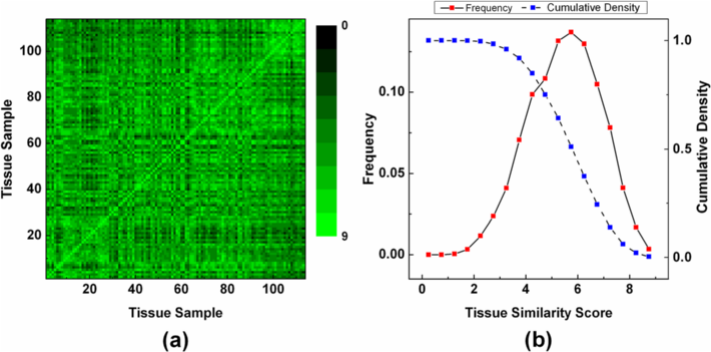
Tissue Morphologic Similarity Scores. **a** Tissue morphologic similarity scores are computed and drawn for all possible pairs of tissue samples. **b** The frequency and cumulative density of similarity scores are plotted. Mid-range scores (5 ~ 6) are mostly dominant, and high scoring (≥8) samples are very rare

For 114 prostate cancer samples, we asked a pathologist (A.K.-B) to score them according to the 9 morphologic criteria. The pathologist was not involved in preparing the tissue samples and kept blind to the previous diagnosis and clinical information of the samples. Provided with the scores for the 9 morphologic criteria, tissue morphologic similarity (*TMS*) was measured for all possible pairs of 114 tissue samples (Fig. 2c and Fig. 3a) and used as the gold standard for training and validating our approach. We noted that *TMS* score, ranging from 0 to 9, is not evenly distributed, and mid-range score (5 ~ 6) is mostly dominant. Notably, only small number of pairs of samples gained a high *TMS* score, e.g., ~2 % of pairs of samples scores ≥8 (Fig. 3b).

### Tissue retrieval system provides good matching cases

To evaluate the tissue retrieval system, we performed *K*-fold cross-validation (*K* = 10; maintaining a sufficient number of tissues in the database). The entire dataset was divided into *K* roughly equal-sized partitions, one partition was left out as “test data” (or queries), the union of the remaining *K – 1* partitions (the “training data”) was used to build the database where top-*T* similar samples are retrieved for each query (*T* = 5). This was repeated *K* times with different choices of the left-out partition. In each repetition, the 2-stage feature selection was carried out on the training data via a cross-validation (5-fold). The average NDCG at rank position *T* of the tissue retrievals for the queries, across all *K* repetitions, was computed to measure the performance of the retrieval. To handle the imbalance of *TMS* scores in the dataset, a roughly balanced training dataset was formed by dividing the entire score range into *P* equal-width partitions (*P* = 10; allocating a sufficient number of tissues per partition in regard to the number of retrieved samples) and randomly taking equal number of samples from each partition. The method was implemented in IDL (tissue segmentation and morphological feature extraction) on 1 1.67GHz Intel Core Duo machine running Windows 7 with 2GB memory and C++ (feature selection and tissue retrieval) on a 2.5GHz Intel Core 2 Duo machine running Redhat Linux 4 with 2GB memory. The average processing time for tissue segmentation and morphological feature extraction is ~8 min per sample, and the tissue retrieval time is ~1 s. The Ranking-SVM training and the feature selection took ~3 s and ~90 min, respectively.

**Fig. 4:**
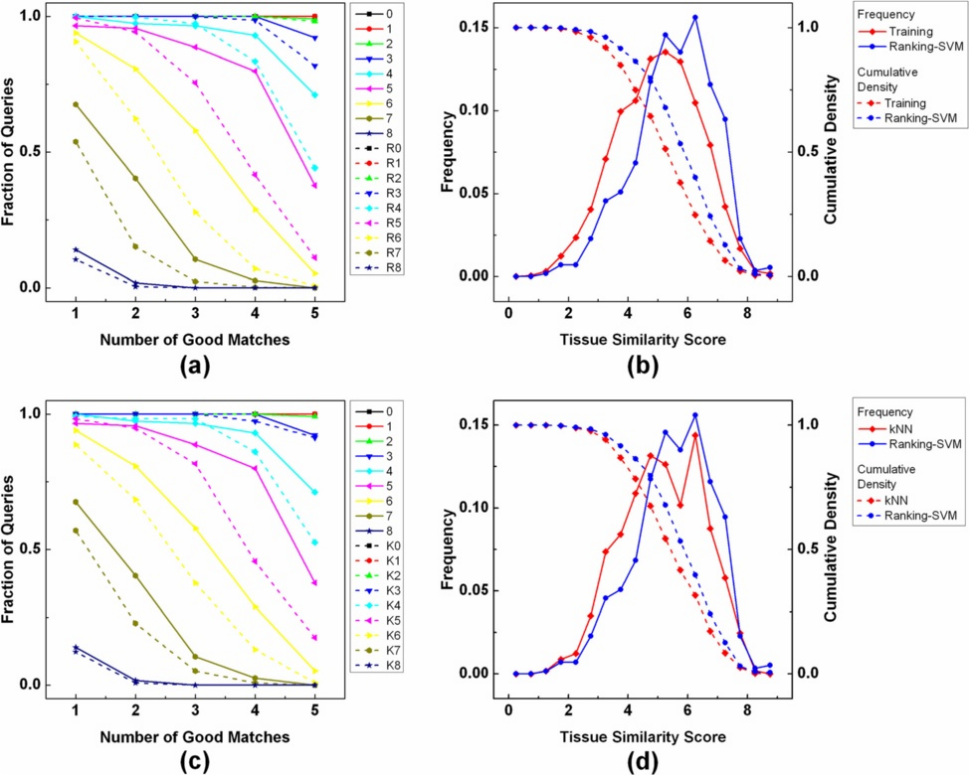
Tissue Retrieval Performance. The number of queries retrieving at least *N*_*G*_ number of good matches by our system (Ranking-SVM), out of *T* retrieved samples, is computed (*N*_*G*_ = 1, …, *T*), and compared to **a** the random chance (R0 ~ R9) and **c** kNN retrieval (K0 ~ K9) obtaining that number of good matching cases. The frequency and cumulative density of similarity scores are plotted for **b** the entire training samples and *T* matching samples by our system, respectively. **d** The frequency and cumulative density are also plotted for kNN retrieval. A good matching case is defined as a pair of samples whose similarity score is ≥ *th*_*s*_, *th*_*s*_ = 0, …, 8. Random chance of retrieving ≥ *N*_*G*_ good matching cases is computed as $$ \Pr \left(X\ge {N}_G\right)={\displaystyle {\sum}_{x\ge {N}_G}\frac{\left(\begin{array}{c}\hfill {N}_{ss}\hfill \\ {}\hfill x\hfill \end{array}\right)\left(\begin{array}{c}\hfill {N}_s-{N}_{ss}\hfill \\ {}\hfill T-x\hfill \end{array}\right)}{\left(\begin{array}{c}\hfill m\hfill \\ {}\hfill T\hfill \end{array}\right)}} $$ where *N*_*S*_ and *N*_*SS*_ denote the number of samples in the database and the number of samples whose *TMS* with the query ≥ *th*_*s*_, respectively

Although we have computed *TMS* scores and used them to train and test the retrieval process, it is unclear what similarity score is sufficient to provide useful information with pathologists when evaluating unknown samples. Setting a threshold *TMS* too high score is unrealistic because there are not enough samples available; as mentioned above, only ~2 % of the training samples have similarity score ≥8 for a query (Fig. 3b). Setting the *TMS* threshold lower is not beneficial to pathologists. We therefore adopted a new data management approach: In order to examine the retrieval performance in a broad sense, we changed a threshold similarity score *th*_*s*_ from 0 to 8, and designated a sample as a good match (or relevant sample) to a query if their similarity score is ≥ *th*_*s*_. Then, we counted the number of good matches (*N*_*G*_) among the retrieved samples for each query and plotted the fraction of the queries retrieving ≥ *N*_*G*_(*N*_*G*_ = 1, …, *T*). *N*_*G*_ among the retrieved samples is equivalent to the fraction of the retrieved samples that are relevant to the query (“precision”). That is, Fig. 4a shows the fraction of the queries achieving a precision level equal to or higher than 0.2, 0.4, 0.6, 0.8, and 1. It is noticeable that ~80 % and ~60 % of the queries retrieving ≥4 and ≥3 good matching cases (or ≥0.8 and ≥0.6 precision) as setting *th*_*s*_ to 5 and 6, respectively. Compared to the random chance of retrieving ≥4 and ≥3 good matches, both were increased by two-fold, and the retrievals were statistically significant (*p-value* <1.0e-10) by a binomial test (Table 2). As shown in Fig. 4b, it was obvious that *TMS* scores of pairs of the query and its top-*T* matching samples are higher than those of pairs of the query and all the samples in the database, especially *TMS* scores are 5 or greater.

**Table 2:** Statistical significance of tissue retrieval

		*th*_*s*_		
		5	6	7
*N*_*G*_	3	101 <0.001	66 <1.e-10	12 <0.0001
	4	91 <1.e-16	33 <1.e-11	3 <0.01
	5	43 <1.e-12	6 <0.001	0 1

The number of queries (*N*_*q*_) retrieving at least *N*_*G*_ number of good matching cases as a good match is defined as *TMS* ≥ *th*_*s*_ and its statistical significance. Assuming the number of good matches follows a binomial distribution, *p-value* is computed as $$ \Pr \left(X\ge {N}_q\right)={\displaystyle {\sum}_{x\ge {N}_q}\left(\begin{array}{c}\hfill m\hfill \\ {}\hfill x\hfill \end{array}\right){p}^x{\left(1-p\right)}^{m-x}} $$ where *p* is a random chance of retrieving ≥ *N*_*G*_ good matches (*TMS* ≥ *th*_*s*_). (top) the number of queries and (bottom) its statistical significance

Moreover, we performed the tissue retrieval by using the k-Nearest Neighbor (kNN) algorithm (k = 5), instead of Ranking-SVM. Examining the number of good matches, Ranking-SVM consistently outperformed kNN; for instance, setting *th*_*s*_ to 5 and 6, Ranking-SVM demonstrated a 1.5-fold increase in the fraction of the queries retrieving ≥4 and ≥3 good matches, respectively (Fig. 4c). We investigated the distribution of *TMS* scores of pairs of the query and top-*T* matching samples by Ranking-SVM and kNN (Fig. 4d). Ranking-SVM showed higher *TMS* scores than kNN (*TMS* score ≥ 5). Further, the retrieval results were evaluated by using NDCG (Table 3). Considering top-*T* matching samples, Ranking-SVM achieved the average NDCG of 0.35, and 0.29 NDCG was obtained by kNN on average. NDCG was computed for the ranking of the entire samples in the database; Ranking-SVM and kNN showed the average NDCG of 0.75 and 0.68 NDCG, respectively.

**Table 3:** Tissue retrieval performance

	Top-*T*	All samples
Ranking-SVM	0.35 ± 0.13	0.75 ± 0.06
kNN	0.29 ± 0.14	0.68 ± 0.06

Data represent average ± standard deviation of NDCG

### TMS score reveals the complicated relationship between tissues

**Fig. 5:**
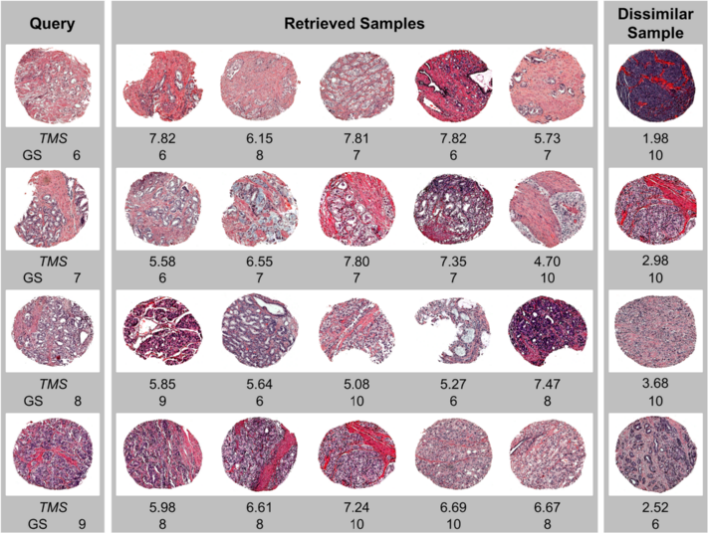
Examples of queries and their matching cases. For each query (*left column*), 5 closest matches are retrieved. The least similar sample is also vshown (*right column*). *TMS* denotes tissue morphologic similarity score for a pair of samples. GS indicates a Gleason sum score which is a sum of predominant and secondary Gleason scores

We examined the utility of *TMS* scores in retrieving similar tissue samples by a visual comparison between tissue H&E images. The relationship between *TMS* score and Gleason sum score was also investigated since Gleason sum score is the only definite information available in prostate pathology today. In Fig. 5, the examples of queries and their matching cases are presented. A pair of samples belonging to the same grade tends to have a (relatively) high *TMS* score, for example, in the second row of Fig. 5, three retrieved samples with Gleason sum score 7 have >6.5 *TMS* score for the query whose Gleason sum score is 7. Other two samples have different Gleason sum score as well as lower *TMS* scores (<5.6). However, high *TMS* scoring sample pairs are not necessarily to be the same grade. In the last row of Fig. 5, none of the retrieved samples are diagnosed with the same Gleason sum score with the query, but their *TMS* scores are generally high. Four of them have >6.6 *TMS* score, of which each has identical scores with the query for at least 4 morphologic criteria except Gleason score, demonstrating the capability of *TMS* scoring system in examining the relationship between tissues beyond the Gleason grading system. These types of relationships between tissue samples can never be retrieved or assessed if an automated system is built solely on the Gleason grading system. Thus, *TMS* scoring system may help to analyze the complicated and complex tissue morphology and to broaden our understanding.

## Discussion

Herein, a tissue retrieval system has been developed and tested for prostate cancer. This approach is particularly well suited for cancer and other diagnostic situations where there are multiple parameters applied to defining a grade. In the system, a database allows pathologists to easily manage and maintain the previous cases and outcomes, and immediate access to them is available due to efficient retrieval algorithm. Accordingly, the performance of tissue retrieval is reliant on both a database and a retrieval process. Hence, further study on matching algorithm, performance measure, and data handling, e.g., data normalization, would be necessary, and a large-scale validation study should be conducted to optimize and stabilize the system for various queries, tasks and users’ demands.

The size of the database may substantially affect the performance of the retrieval system. In tissue retrieval, it is assumed that the database contains enough number of similar samples to any kind of query. That is, the retrieval system will benefit from the large-scale database, including a variety of patterns of tissue samples from multiple institutions. The retrieval system with the large-scale database will not only serve for various queries and tasks but also improve and stabilize *TMS* scores. The similarity score for a criterion between two samples is dependent on the number of samples between them according to the criterion. The distribution of the samples will become more realistic, leading to the more accurate and reliable similarity measure. Moreover, scoring tissue samples by multiple pathologists will further aid in improving *TMS* scores. However, with the limited size of the database, the distribution of *TMS* score for one query differs from another (Fig. 3a). Some may have many high scoring sample pairs, but some may have few of them. In the latter cases, the retrieval system may return the most similar samples, i.e., the retrieval is valid and useful, but it is a seemingly bad retrieval due to relatively lower *TMS* score. The overall distribution of *TMS* score also affects the retrieval. In our study, a limited number of tissue sample pairs show a high or low *TMS* score (Fig. 3b), i.e., it is likely that the system retrieves tissue samples owning mid-range *TMS* scores. In fact, as we trained Ranking-SVM on the entire training dataset, i.e., without balanced training, less number of samples owning higher *TMS* scores was retrieved for the query (Additional file 1: Figure S1), for example, *TMS* score ≥6. Accordingly, taking a roughly balanced subset of the training dataset is a valid decision and helps to provide a more effective and robust retrieval process.

Gleason grades in the dataset are not evenly distributed. A lack of a sufficient number of samples per grade may result in a loss of information of certain patterns in prostate cancer. However, the imbalance of the distribution in this study is not likely to have a significant impact on the retrieval system. The system is still able to retrieve matching cases from the database. A high *TMS* score does not indicate that a sample pair has the same grade. The effect of each grade on the retrieval system may be further studied to improve and stabilize the retrieval system.

We only retrieved the 5 closest samples to a query. The more samples we retrieve, the higher probability the system provides well matched cases with pathologists. However, retrieving many samples (e.g., >10) will be burden to pathologists due to additional time and effort to decide what samples are relevant and useful. Hence, providing the most similar samples would be more helpful and effective. It necessitates little time and work from pathologists to judge on the retrieved samples, however deliver good matches. We note that if a pathologist would like to retrieve more or fewer samples from the database, then the retrieval system (Ranking-SVM) should be re-trained by adjusting the number of retrievals. If more samples are added to the database, then the whole system should be re-trained (or updated) by computing TMS scores and morphological features and constructing a new Ranking-SVM. Moreover, as one or more morphological properties are of interest to a pathologist, the similarity score can be re-computed and used to train the retrieval system. The pathologist may indicate that certain matches were better than others, resulting in an updating of the database (e.g., changing TMS score) and matching algorithms as needed. The updating may be conducted in real-time. Therefore, the system is potentially adaptable to users’ demand and purpose.

The 9 morphological criteria were manually scored by a pathologist and used to measure *TMS* score. Like Gleason grading, it is still a qualitative measure. Based on the qualitative measure, the pathologist categorizes (or scores) tissue samples per criterion. It is well known that such qualitative measure is subject to inter- and intra-observer variability, i.e., likely mis-score (or mis-classify) tissue samples, in particular for the borderline cases. Poor scoring (or mis-scoring), in our study, will disrupt the similarity measure. However, the impact of mis-scoring on the retrieval system may not be as significant as that of Gleason grading. Mis-scoring in Gleason grading may give rise to a totally different pattern and outcome prediction. Unlikely, *TMS* score is a combined measure of the 9 different properties and varies in a continuous fashion. Some mis-scorings of the 9 criteria clearly affect the similarity measure but may not cause a complete change in the tissue similarity. Nevertheless, a follow-up study is desirable to examine the influence of mis-scorings among the 9 criteria on the similarity measure and the tissue retrieval performance.

## Conclusions

We have presented an efficient and effective tissue management and decision-support system. *TMS* score offers an alternate means of assessing tissue characteristics and similarities as well as developing and testing computerized methods. Next steps in development would be the validation and application of this system with additional users. The system can be applied to a diversity of diagnostic entities in histopathology. The approach is adaptable in scale, including reference dataset, scoring metrics and matches presented to the pathologist. We anticipate that this approach will open a new direction for the development of automated methods for cancer pathology.

## Additional file

Additional file 1: Supplementary material. (PDF 437 kb)
