# Supplementary material for: Automated prostate tissue referencing for cancer detection and diagnosis
Source: BMC Bioinformatics. 2016 Jun 1;17:227. doi: 10.1186/s12859-016-1086-6 (PMC4888626; doi:10.1186/s12859-016-1086-6)
Supplement: Supplementary file 1 — Supplementary material. (PDF 437 kb) [file 12859_2016_1086_MOESM1_ESM.pdf]

Supplementary Information for

AUTOMATED PROSTSTATE TISSUE REFERENCING FOR CANCER DETECTION AND DIAGNOSIS

Jin Tae Kwak[1], Stephen M. Hewitt[2], André Alexander Kajdacsy-Balla[3], Saurabh Sinha[4,*], Rohit Bhargava[5,*]

[1]Department of Computer Engineering, Sejong University, Seoul 05006, Korea

[2]Tissue Array Research Program, Laboratory of Pathology, Center for Cancer Research, National Cancer Institute, National Institutes of Health, Bethesda, MD 20850, USA

[3]Department of Pathology, University of Illinois at Chicago, Chicago, IL 60612 USA

[4]Department of Computer Science, University of Illinois at Urbana-Champaign, Urbana, IL 61801, USA

[5]Beckman Institute for Advanced Science and Technology, Department of Bioengineering, Department of Mechanical Science and Engineering, Electrical and Computer Engineering, Chemical and Biomolecular Engineering and University of Illinois Cancer Center, University of Illinois at Urbana-Champaign, Urbana, IL 61801, USA

[*] Corresponding author

The detailed description of a morphological feature extraction process and a retrieval algorithm and tissue retrieval results without balanced training (Figure S1) are described and presented below.

Morphological feature extraction

Previously, 17 quantities to describe structural properties of prostate tissue were defined and used to detect cancer tissue [1]. 8 of them are epithelium related features: 1) Size of epithelial cells 2) Size of a nucleus 3) Number of nuclei 4) Distance to lumen 5) Distance to epithelial cell boundary 6) Number of isolated nuclei 7) Fraction of distant nuclei 8) Entropy of nuclei spatial distribution. The rest of 9 quantities are lumen-related features: 1) Size of a lumen 2) Number of lumens 3) Lumen roundness 4) lumen distortion 5) Lumen minimum bounding circle ratio 6) Lumen convex hull ratio 7) Symmetric index of lumen boundary 8) Symmetric index of lumen area 9) Spatial association of lumens and cytoplasm-rich regions. Additionally, we have defined 9 quantities describing characteristics of epithelium, stroma, lumens, and glands:

1) Number of stroma cells: Number of stroma pixels in a tissue.

2) Minimum lumen distance: Minimum distance between lumens.

3) Minimum gland distance: Minimum distance between glands. To find glands in a tissue, we first find neighboring nuclei for each lumen. Neighboring nuclei to a lumen are the ones present within the epithelial cells next to the lumen. Then, we find a subset of the neighboring nuclei C_{sub} which satisfy the following condition:

$$\forall \mathrm{c} \in \mathrm{C}_{sub}: \mathrm{Distance}(c,l) \leq AVG\left(\mathrm{Distance}(\mathrm{C}_{sub},l)\right) + m * STD\left(\mathrm{Distance}(\mathrm{C}_{sub},l)\right)$$

where l is a lumen, $\mathrm{Distance}(c,l)$ denotes the distance from a nucleus c to a lumen l, and AVG(•) and STD(•) indicates the average and standard deviation of •. Initially, m is set to 1.5. At each iteration, nuclei which do not satisfy the criteria are eliminated for further consideration and

m is increased by 0.01. Fitting an ellipse to the subset of the neighboring nuclei gives an estimate of a gland for the lumen.

4) Ratio of lumen to epithelial cells: Ratio of the number of lumen pixels to the number of epithelial pixels in a tissue.

5) Ratio of epithelial cells to stroma cells: Ratio of the number of epithelial pixels to the number of stroma pixels.

6) Ratio of cell separation: Ratio of the number of separated epithelial nuclei to the total number of epithelial nuclei. Epithelial cells are designated as separated cells if their size < 500 pixels and >90% of their boundary is next to stroma cells.

7) Ratio of sheets of cells: Ratio of the number of nuclei which are not associated with any gland and do not belong to the separated cells to the total number of nuclei.

8) Degree of cell dispersion: Degree of dispersion of a cell type can be measured by variance-to-mean ratio (VMR) [2]. It is defined as VMR=$\frac{\sigma^2}{\mu}$ where μ is the average number of the pixels labeled with a cell type and σ^2 is the variance of the number of the pixels labeled with the cell type. VMR is separately computed for epithelial and stroma cells.

9) Spatial autocorrelation of cells: To compute spatial autocorrelation of a cell type, we adopt two measures: Moran's I and Greary's C. Moran's I [3] can be computed as follows:

$$I = \frac{n}{\sum_i^n \sum_j^n w_{ij}} \frac{\sum_i^n \sum_j^n w_{ij} \left(y_i - \bar{y}\right)\left(y_j - \bar{y}\right)}{\sum_i^n \left(y_i - \bar{y}\right)^2}$$

where n is the number of the pixels assigned to the cell type, w is a random variable representing a weight associated with each pair of the pixels, y is a random variable for the number of adjacent pixels containing the same cell type, $\bar{y}$ is the average of the number of adjacent pixels. For simplicity, w_{ij} =1 if i and j are adjacent and w_{ij} =0 otherwise. Similarly, Greary's C [4] can be calculated as follows:

$$C = \frac{n-1}{\sum_i^n \sum_j^n w_{ij}} \frac{\sum_i^n \sum_j^n w_{ij} \left(y_i - y_j\right)^2}{\sum_i^n \left(y_i - \bar{y}\right)^2}.$$

Prior to compute spatial autocorrelation of a cell type, for each pixel, the number of the adjacent pixels labeled with the same cell type is computed. These counts are used to measure spatial autocorrelation. Both Moran's *I* and Greary's C are computed for epithelial cells and stroma cells, respectively.

For each of 26 quantities, we measure "global" and "local" features. To compute, "global" features, we employ AVG, STD, and sum total (TOT) of the quantities. "Local" features are calculated by sliding a rectangular window (*N*x*N* pixels) throughout a tissue sample. For each window, AVG and/or TOT of the quantities are computed, and then STD or MIN/MAX of the AVG and/or TOT values over all windows become "local" features. 5 different sizes of the window (*N*=20, 60, 100, 140, 180) are applied, and the "local" features are computed for each.

Ranking-SVM

Given a training dataset $\{(x_i, y_i)\}_{i=1}^{m}$ with a feature vector $x_i \in \Re^n$ and a (class) label $y_i \in \{-1, +1\}$, a classification support vector machine (SVM) learns a separating hyperplane which maximizes the margin between support vectors representing different classes. In Ranking SVM [5], a label y_i denotes an ordering preference or a rank but a category, i.e., $\forall y_i \in \Re$ and a complete ranking can be made among the labels. It seeks to learn a function $f \in F$ satisfying the following relations for any pair of data points:

$$y_i > y_j \Leftrightarrow f(x_i) > f(x_j).$$

Constructing such function can be formulated as follows:

$$\min_{w, \xi_{ij} \geq 0} \frac{1}{2} w^T w + \frac{C}{|P|} \sum_{(i,j) \in P} \xi_{ij}$$
$$s.t.\ \forall (i, j) \in P:\ w^T (x_i - x_j) \geq 1 - \xi_{ij}$$

where C is a tradeoff between training error and model complexity and ξ_{ij} is a slack variable. Intuitively, the function aims at minimizing the number of swapped pairs of training data points in terms of their desired rankings [5]. Interestingly, optimizing the formula is, in fact, equivalent to that of a classification SVM as pairwise difference vectors $(x_i - x_j)$ are provided.

In our system, the label is given by a tissue similarity score between a pair of tissue samples, and a feature vector is generated by the difference vector between the feature vectors of the pair. In other words, a pair of tissue samples forms one instance to train Ranking-SVM, and it attempts to learn the ordering preference between the pairs of samples. Thus, as a query is given to our

system, we generate an instance for each pair of a query and a tissue sample in the database, and the rankings of the entire instances are predicted by Ranking-SVM. The samples which result in the top-*T* ranking instances with the query are designated as most similar samples and provided with pathologists.

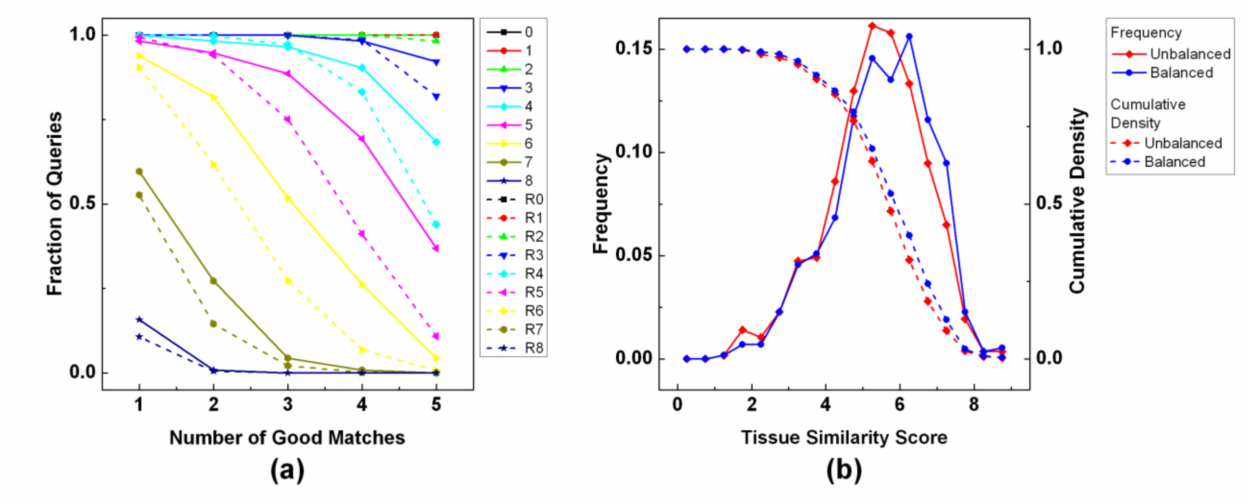

Figure S1. Queries retrieving good matching cases without balanced training. (a) The number of queries retrieving at least N_G number of good matches, out of T retrieved samples, is computed (N_G=1,…,T), and compared to the random chance (R0~R9) of retrieving that number of good matching cases. (b) The frequency and cumulative density of similarity scores are plotted as retrieval process is trained on balanced training dataset and unbalanced training dataset, respectively. A good matching case is defined as a pair of samples whose similarity score is $\geq th_s$, th_s =0,…,8. Random chance of retrieving $\geq N_G$ good matching cases is computed as $Pr(X \geq N_G) = \sum_{x \geq N_G} \frac{\binom{N_{SS}}{x}\binom{N_S - N_{SS}}{T-x}}{\binom{m}{T}}$ where N_S and N_{SS} denote the number of samples in the database and the number of samples whose *TMS* with the query $\geq th_s$, respectively.

REFERENCES

1. Kwak J, Hewitt S, Sinha S, Bhargava R. Multimodal microscopy for automated histologic analysis of prostate cancer. *BMC Cancer* 2011; 11(1):62.

2. Cox DR. *The statistical analysis of series of events*, Vol., Methuen: London, 1966.

3. Moran PA. Notes on continuous stochastic phenomena. *Biometrika* 1950; 37(1-2):17-23.

4. Geary RC. The Contiguity Ratio and Statistical Mapping. *The Incorporated Statistician* 1954; 5(3):115-46.

5. Joachims T. Training linear SVMs in linear time. *Proceedings of the Proceedings of the 12th ACM SIGKDD international conference on Knowledge discovery and data mining*2006: ACM.
